# Directionally aligned nanofibrous polycaprolactone/gelatin scaffolds as durable scleral implants for long‐term ocular reconstruction

**DOI:** 10.1002/btm2.70196

**Published:** 2026-11-10

**Authors:** Ikhyun Jun, Seungmin Kang, Taehyeon Kim, Youngbin Seo, Hae‐sol Shin, Sejin Myeong, David Myung, Kyoung Yul Seo, Won‐Gun Koh

**Affiliations:** ^1^ The Institute of Vision Research, Department of Ophthalmology Yonsei University College of Medicine Seoul South Korea; ^2^ The Corneal Dystrophy Research Institute, Department of Ophthalmology Yonsei University College of Medicine Seoul South Korea; ^3^ Department of Chemical and Biomolecular Engineering Yonsei University Seoul South Korea; ^4^ Spencer Center for Vision Research, Byers Eye Institute, Department of Ophthalmology Stanford University Palo Alto California USA; ^5^ Department of Chemical Engineering Stanford University Stanford California USA

**Keywords:** aligned PCL/gelatin fiber, electrospun fiber, fiber orientation, scleral implant

## Abstract

Scleral defects caused by pathological conditions or surgical complications remain a major challenge in ophthalmology owing to the lack of durable implantable materials. In this study, we developed electrospun polycaprolactone/gelatin (PCL/GE) nanofibrous scaffolds with controlled fiber orientation for long‐term scleral reconstruction. Three scaffold architectures—random, radially aligned, and uniaxially aligned—were fabricated and benchmarked against Ologen™, a clinically used collagen matrix. The scaffolds exhibited an average fiber diameter of approximately 1 μm, high porosity (96.3%–96.9%), and tensile properties comparable to those reported for native rabbit and human sclera. In vitro, anisotropic scaffolds significantly enhanced fibroblast proliferation, directional organization, and collagen type I production compared with random scaffolds. Following enzymatic degradation for 6 weeks, the PCL/GE scaffolds retained approximately 77% of their initial mass, compared with approximately 70% for Ologen™. In a rabbit scleral defect model, anisotropic scaffolds promoted smoother surface healing and reduced peri‐implant inflammation at 6 weeks without significant changes in intraocular pressure or pro‐inflammatory cytokine expression. At 24 weeks, all PCL/GE scaffolds remained structurally intact, whereas Ologen™ had completely degraded, resulting in choroidal exposure. Histological analysis further revealed that the radially aligned scaffold produced the thinnest fibrotic capsule and more favorable tissue integration than the random scaffold. These findings demonstrate that electrospun PCL/GE nanofibrous scaffolds provide mechanical compatibility, long‐term structural stability, and excellent biocompatibility, highlighting their potential as durable implants for scleral reconstruction.

## Graphical abstract

This study demonstrates the potential of aligned electrospun PCL/gelatin nanofibrous scaffolds as stable, biocompatible, and cosmetically favorable implants for scleral reconstruction by promoting organized tissue regeneration and outperforming conventional materials over 24 weeks.

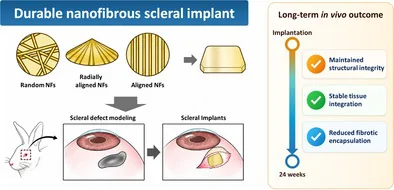

**Translational Impact Statement**

Scleral defects remain a significant clinical challenge because currently available graft materials often lack sufficient long‐term durability. We developed electrospun polycaprolactone/gelatin nanofibrous scaffolds with anisotropic fiber architectures that provide directional structural cues, support fibroblast integration, and promote favorable tissue organization. In a rabbit scleral defect model, these scaffolds showed good in vivo biocompatibility with limited inflammatory and fibrotic responses, while maintaining structural integrity and stable tissue integration for up to 24 weeks. In contrast, the clinically used collagen matrix Ologen™ was completely degraded by 24 weeks, resulting in loss of long‐term defect coverage. These findings highlight the potential of anisotropic PCL/gelatin nanofibrous scaffolds as durable implants for long‐term scleral reconstruction.

## 1 INTRODUCTION

The sclera, a dense fibrous connective tissue that constitutes approximately five‐sixths of the outer layer of the human eye, serves as a critical structural component that maintains ocular shape and protects intraocular structures by withstanding intraocular pressure (IOP).^1^, ^2^ Histologically, the sclera is predominantly composed of type I collagen fibers arranged in a complex laminar architecture, along with proteoglycans, fibroblasts, elastin, and vascular structures that collectively provide mechanical strength and flexibility.^1^, ^2^, ^3^

Scleral defects represent a significant clinical challenge in ophthalmology, resulting from various pathological conditions such as scleromalacia, necrotizing scleritis, and infectious scleritis, or as complications following ocular surgeries such as pterygium excision, retinal detachment repair, and glaucoma filtering procedures.^4^, ^5^, ^6^ The incidence of scleral necrosis following ocular surgery has been reported to range from approximately 0.2% to 4%, depending on surgical technique and adjunctive therapies. These defects can lead to exposure of the underlying uveal tissue, compromising both structural integrity and cosmetic appearance owing to the characteristic darkened discoloration.^7^ Furthermore, the altered surface contour may result in tear film instability, dellen formation, chronic irritation, and patient discomfort.^8^ If left untreated, severe scleral thinning can progress to perforation, potentially leading to vision‐threatening complications.^9^

Current treatment strategies for scleral reconstruction have inherent limitations that highlight the need for improved therapeutic approaches. Allogeneic scleral grafts, which offer excellent anatomical similarity and biocompatibility, are severely limited by donor tissue availability, risk of immune rejection, and potential for disease transmission.^6^, ^10^, ^11^ Although autologous grafts eliminate immunological concerns, they create secondary donor site morbidity and may be insufficient for large defects.^12^, ^13^ Amniotic membrane transplantation provides anti‐inflammatory benefits and promotes epithelialization, but lacks the mechanical strength required for long‐term structural support in load‐bearing applications.^14^, ^15^

Commercially available crosslinked collagen matrices, such as Ologen™, have gained clinical acceptance for temporary coverage in glaucoma surgery and scleral defect repair owing to their biocompatibility and ease of handling.^16^, ^17^, ^18^ However, these materials suffer from rapid biodegradation, typically resorbing within 3–6 months, which often results in inadequate long‐term protection, tissue re‐exposure, and suboptimal cosmetic outcomes.^19^, ^20^, ^21^ This rapid degradation timeline is particularly problematic for scleral applications where sustained structural support is essential for proper healing and prevention of complications. In addition to its biological limitations, the clinical use of Ologen™ has been further constrained by recent supply and availability issues in certain regions.

Tissue‐engineering approaches using polymer‐based scaffolds have emerged as promising alternatives to address these clinical limitations. Biodegradable polymers, such as polycaprolactone (PCL) and poly(lactide‐co‐glycolide) (PLGA), offer several advantages, including controllable degradation kinetics, predictable mechanical properties, and the ability to avoid immunogenic responses associated with biological materials.^22^, ^23^, ^24^ However, conventional bulk hydrophobic polymer scaffolds often lack the architectural complexity and flexibility required for optimal tissue integration.^25^, ^26^

Electrospinning has emerged as an effective fabrication technique for creating nanofibrous scaffolds that closely mimic the hierarchical structure of the native extracellular matrix (ECM).^27^, ^28^, ^29^, ^30^, ^31^, ^32^ This technique enables precise control over fiber diameter, orientation, and scaffold architecture, thereby creating biomimetic environments that regulate cellular behavior and tissue regeneration.^33^, ^34^ In particular, electrospinning can generate nanofibrous scaffolds with random, radially aligned, or uniaxially aligned fiber architectures. These distinct fiber organizations differentially regulate cell adhesion, migration, alignment, and extracellular matrix deposition. While aligned fibers provide directional guidance for cell organization, radially aligned fibers promote centripetal cell migration, highlighting fiber orientation as an important design parameter in tissue engineering.^33^, ^35^, ^36^ Extensive research has explored electrospun nanofibers for ophthalmic applications, including corneal regeneration, glaucoma drainage implants, and ocular drug delivery systems; however, their application in scleral implants remains largely unexplored.^37^, ^38^, ^39^, ^40^, ^41^ Given that the sclera is an anisotropic tissue whose mechanical function depends on the highly organized arrangement of collagen fibers, controlling fiber orientation may provide a promising strategy for guiding organized scleral tissue regeneration and maintaining structural integrity.

In this study, we developed electrospun polycaprolactone/gelatin (PCL/GE) nanofibrous scaffolds specifically engineered for long‐term scleral reconstruction. To investigate the impact of the scaffold microarchitecture on tissue integration and regenerative outcomes, we fabricated three distinct fiber orientations (random, radially aligned, and aligned) via controlled electrospinning techniques. PCL was selected because of its excellent mechanical durability, slow hydrolytic degradation, and biocompatibility, whereas gelatin was incorporated to enhance cell–material interactions and provide ECM‐like biochemical cues. As gelatin gradually degrades during tissue regeneration, the remaining PCL nanofibrous network continues to maintain the structural integrity of the scaffold while supporting tissue remodeling. This complementary design strategy enables the scaffold to provide both early biological activity and long‐term mechanical support, making it suitable for scleral tissue regeneration.

We hypothesized that fiber alignment would modulate fibroblast behavior, influence the quality and organization of tissue regeneration, and ultimately lead to improved clinical outcomes compared with randomly oriented architectures. To establish clinical relevance and benchmark performance, we compared these novel scaffolds with Ologen™, a widely used crosslinked collagen matrix that represents the current clinical standard despite its limitations in long‐term retention. We performed a comprehensive evaluation of physicochemical properties, in vitro cellular responses, and long‐term in vivo performance in a standardized rabbit scleral defect model. The objective was to determine whether fiber‐aligned PCL/GE scaffolds can provide a more durable, biocompatible, and cosmetically favorable alternative for sustained scleral reconstruction.

## 2 MATERIALS AND METHODS

### 2.1 Materials

Unless otherwise stated, all chemicals and solvents were of analytical grade and used as provided by the manufacturers. PCL (molecular weight 80 kDa), 2,2,2‐trifluoroethanol (TFE), bovine type I gelatin (GE), glutaraldehyde, and dimethyl sulfoxide (DMSO) were purchased from Sigma‐Aldrich (Milwaukee, WI, USA). 3‐(4,5‐Dimethylthiazol‐2‐yl)‐2,5‐diphenyltetrazolium bromide (MTT) was purchased from Invitrogen Corp. (Carlsbad, CA, USA). NIH/3T3 cells (mouse embryonic fibroblast, strain: NIH/Swiss, embryo; official name: NIH/3T3; RRID: CVCL_0594) were purchased from Fisher Scientific (Waltham, MA, USA; Cat. No. 502383450), originally supplied by ATCC (CRL‐1658), in 2022. The cell line is authenticated by ATCC prior to distribution. Although we did not perform additional mycoplasma testing in our laboratory, NIH/3T3 cells from ATCC/Fisher are certified mycoplasma‐free and no abnormal growth characteristics were observed during culture.

### 2.2 Fabrication of electrospun PCL/GE NFs

The PCL/GE nanofibrous scaffolds were fabricated via electrospinning. Initially, a PCL/GE solution was prepared by dissolving polycaprolactone (PCL) and gelatin (GE) in 2,2,2‐trifluoroethanol (TFE) at a concentration of 5% w/v for each polymer. The solution was maintained at 37°C for 24 h to ensure the complete dissolution of both components. Prior to electrospinning, the solution was equilibrated at room temperature and vortexed thoroughly to achieve homogeneity. For the electrospinning process, the polymer solution was loaded into a 10 mL syringe equipped with a 20‐gauge needle. The PCL/GE solution was dispensed into the collector at a constant flow rate of 1 mL/h by using a syringe pump. The collector was connected to the negative electrode and grounded, whereas the syringe needle was connected to the positive electrode. The working distance between the needle tip and collector was maintained at 8 cm with an applied voltage of 12 kV.

To fabricate scaffolds with different fiber orientations, we employed three distinct collection methods, as illustrated in Figure 1. Random nanofibers (Random NFs) were produced using conventional electrospinning with a stationary flat collector plate. Radially aligned nanofibers (Radially aligned NFs) were generated using a specially designed collector system with pins and rings arranged to create an electric field that directed the fibers in a radial pattern from the center to the periphery. Linearly aligned nanofibers (Aligned NFs) were fabricated using a high‐speed rotating cylindrical collector operated at 900 rpm. The PCL/gelatin solution (1:1 ratio) was used as the common precursor for all three scaffold types while maintaining consistent electrospinning parameters, including voltage, flow rate, and working distance across all collection methods. The electrospinning parameters for each scaffold architecture are summarized in Table S1. Based on their fiber orientation, the scaffolds were categorized into three groups: Random, Radially aligned, and Aligned.

**FIGURE 1:**
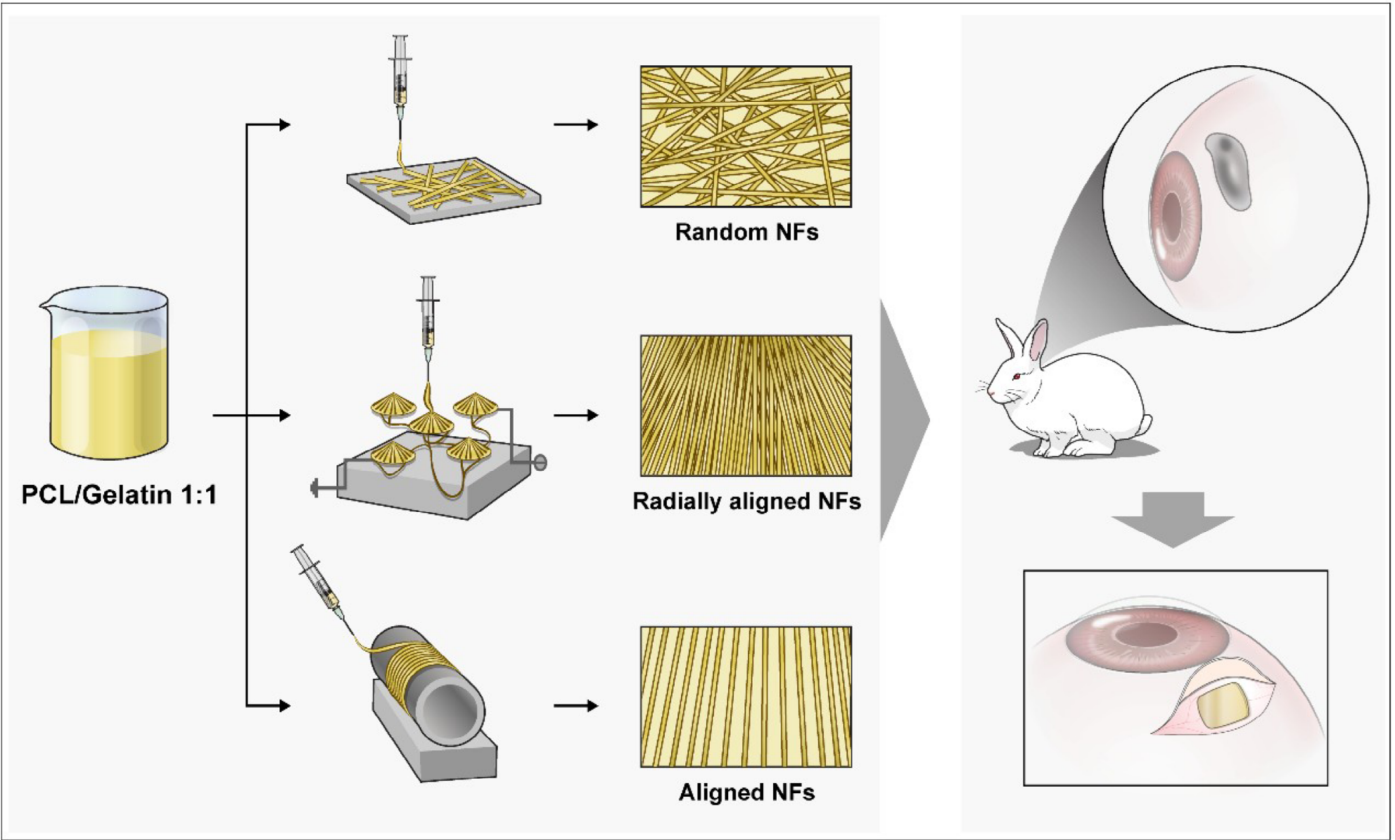
Schematic illustration of the fabrication and orientation control of electrospun PCL/GE nanofibrous scaffolds for scleral reconstruction in rabbit scleral defect model.

The fabricated nanofibrous scaffolds were carefully removed from the collector and subsequently cross‐linked in glutaraldehyde vapor (2% v/v in deionized water) for 24 h under controlled conditions. After crosslinking, the scaffolds were hydrated and thoroughly washed with PBS to remove residual unreacted glutaraldehyde. Prior to biological experiments, the scaffolds were sterilized by exposure to ultraviolet (UV) irradiation for 30 min under aseptic conditions.

### 2.3 Characterization of electrospun PCL/GE NFs

The morphology and orientation of the PCL/GE NFs in the scaffold were examined using field‐emission scanning electron microscopy (FE‐SEM). Before imaging, the samples were sputter‐coated with a thin layer of gold to enhance their conductivity. SEM images were obtained using a JEOL‐7610F‐Plus instrument (JEOL Ltd., Tokyo, Japan) at an accelerating voltage of 10 kV. The fiber diameter and orientation distribution were quantitatively analyzed using the software ImageJ (National Institutes of Health, USA) with the OrientationJ plugin.

The in vitro degradation of the scaffolds was evaluated by monitoring the residual mass over time. Pre‐weighed scaffold samples (approximately 20 mg, *n* = 3 for each group) were immersed in 10 mL of phosphate‐buffered saline (PBS, pH 7.4) containing 0.2 mg/mL Lysozyme in 30 mL vials and incubated at 37°C for 6 weeks. At predetermined time points (weekly intervals), the samples were removed from the PBS, gently rinsed with deionized water, and dried in a vacuum oven at 37°C for 24 h before weighing. The percentage of remaining mass was calculated using the equation: Mass remaining (%) = (*W*_*t*_/*W*_0_) × 100, where *W*_*t*_ is the dry weight at time *t* and *W*_0_ is the initial dry weight. To evaluate the structural integrity and preservation of fiber orientation during degradation, additional scaffold samples were collected after 1, 2, 3, and 4 weeks of enzymatic degradation and analyzed by SEM following the procedure described above. The PBS solution was replaced weekly with fresh PBS to maintain a constant pH.

The chemical structures of PCL, gelatin, and PCL/gelatin nanofibrous scaffolds were characterized using Fourier transform infrared spectroscopy (FTIR) with an FT‐IR spectrometer (VERTEX 70, Bruker, MA, USA). The crystalline properties of PCL, gelatin, and PCL/gelatin nanofibrous scaffolds were analyzed using high‐resolution X‐ray diffraction (HR‐XRD) (SmartLab, Rigaku, Tokyo, Japan).

The tensile mechanical properties of the electrospun scaffolds were evaluated using a universal testing machine (UTM, Mecmesin, Slinfold, West Sussex, UK). For the radially aligned and aligned scaffolds, tensile loading was applied parallel to the principal fiber direction, corresponding to the implantation orientation (fibers perpendicular to the corneal limbus). Young's modulus was determined at 8% strain to enable comparison with previously reported values for native rabbit and human sclera, and the ultimate tensile stress was recorded at failure.^42^

The porous structure of the electrospun scaffolds was quantitatively characterized using mercury intrusion porosimetry (MIP) (AutoPore V, Micromeritics, Norcross, GA, USA). Theoretical porosity, pore volume, pore surface area, and pore‐size distribution were determined according to the manufacturer's instructions.

### 2.4 Cell culture

NIH/3T3 fibroblast cells were cultured in T75 cell culture flasks containing high‐glucose Dulbecco's Modified Eagle Medium (DMEM) supplemented with 10% (v/v) fetal bovine serum and 1% penicillin–streptomycin (10,000 U/mL). Cells were maintained under standard culture conditions (37°C, 5% CO_2_, 95% humidity) in a humidified incubator. The culture medium was refreshed every 3 days, and cells were subcultured twice weekly at approximately 80% confluence using 0.25% trypsin–EDTA solution. For cell seeding experiments, circular PCL/GE NF scaffolds (diameter 15 mm) were placed in 12‐well tissue culture plates and sterilized under UV irradiation for 30 min. Prior to cell seeding, the scaffolds were pre‐wetted with serum‐free DMEM for 2 h to enhance cell attachment. Cell concentration was determined using a hemocytometer, and the cell suspension was adjusted to achieve the desired seeding density. For all experiments, 1.0 × 10^4^ cells in 100 μL medium were carefully seeded onto the center of each scaffold, followed by incubation for 2 h to allow initial cell attachment before adding 2 mL of complete medium to each well.

### 2.5 Cell proliferation and morphological analysis

Cell proliferation and orientation were assessed using the MTT assay and fluorescence live/dead staining. NIH/3T3 cells (1.0 × 10^4^) were seeded on each PCL/GE NF scaffold and cultured for 1, 3, 5, and 7 days. For the MTT assay, the culture medium was removed and the cells were rinsed twice with DPBS. MTT solution (0.5 mg/mL in medium) was added to each well and incubated for 1 h at 37°C. The solution was then removed, and DMSO was added to dissolve the formazan crystals. The absorbance was measured at 540 nm using a microplate reader (Molecular Devices, San Jose, CA, USA). Cell viability and orientation were evaluated using a live/dead assay kit (L‐7013; Invitrogen). The staining solution was prepared by adding calcein AM (20 μL) and ethidium homodimer‐1 (20 μL) to 10 mL of DPBS. After the culture medium was removed and rinsed with DPBS, cells were incubated with the staining solution for 20 min at 37°C. Fluorescence images were captured using an IX53 fluorescence microscope (Olympus Corp.), and cell orientation distribution was analyzed using ImageJ (1.53e).

To evaluate the potential cytotoxicity of scaffold components and degradation‐related leachables, an extract‐based cytotoxicity assay was performed in accordance with ISO 10993 guidelines. Nanofibrous scaffolds were incubated in cell culture medium at an extraction ratio of 6 cm^2^/mL for 24 h to obtain the eluate. Cells were seeded in 6‐well plates at a density of 1.0 × 10^5^ cells per well and allowed to attach. The medium was then replaced with eluate‐containing medium, followed by an additional 48 h incubation. Cell viability was subsequently assessed using a CCK‐8 assay according to the manufacturer's instructions.

### 2.6 Soluble collagen production assay

Collagen type I production using NIH/3T3 fibroblasts cultured on different PCL/GE NF scaffolds was quantified using a Sircol soluble collagen assay kit (Biocolor Ltd., Newtown Abbey, UK). Cell culture supernatants were collected after 3 and 7 days of culturing. For each sample, 1 mL of the supernatant was mixed gently with 1 mL of Sircol dye reagent and incubated for 30 min at room temperature with gentle shaking to allow collagen–dye binding. The samples were then centrifuged at 13,000 × g for 10 min to pellet the collagen–dye complex and the unbound dye‐containing supernatant was carefully discarded. The pellet was resuspended in 1 mL of an alkali reagent and vortexed until completely dissolved. Absorbance was measured at 556 nm by using a microplate reader (Molecular Devices), and the collagen concentration was determined using a standard curve prepared with known concentrations of the collagen standard provided in the kit.

### 2.7 Animals and experimental design

All animal experiments were approved by the Institutional Animal Care and Use Committee of Yonsei University College of Medicine (IACUC 2021‐0327) and performed in accordance with the Yonsei Medical Center Animal Research Guidelines complying with AAALAC standards. A total of 44 female New Zealand White rabbits (2.5–3.5 kg) were acclimatized for 1 week under standard laboratory conditions with ad libitum access to food and water; only female rabbits were used to minimize biological variability and ensure consistency in surgical assessment.

For the main study cohort, 32 rabbits were randomly assigned to evaluate four types of implants—electrospun PCL/GE scaffolds with random, radially aligned, or linearly aligned fiber orientations, and Ologen™—at two postoperative time points: 6 weeks (*n* = 4 per group; total 16) and 24 weeks (*n* = 4 per group; total 16), with sample sizes supported by power analysis (alpha = 0.05, power = 0.80). To further evaluate safety and acute inflammatory responses, an additional cohort of 12 rabbits (*n* = 3 per group) was included to compare the three PCL/GE scaffold types against a surgical control group that underwent the same procedure without implantation. In this safety cohort, intraocular pressure (IOP) was measured using a rebound tonometer Icare® TONOVET Plus (Icare Finland Oy, Helsinki, Finland) before and 6 weeks after surgery, followed by qPCR and histological analysis of inflammatory and fibrotic markers.

All animals were randomly assigned to their respective groups, and blinding was not performed during the surgical procedures or postoperative assessments, as the physical characteristics of the different implant types were readily distinguishable to the investigators. However, to minimize potential bias, objective measurement protocols were strictly followed. The sample size was determined based on a power analysis to ensure sufficient statistical sensitivity while adhering to the ‘3Rs’ principle of animal research. For the main study, a power analysis using G*Power software (version 3.1.9.7) indicated that a sample size of *n* = 4 per group was sufficient to detect a large effect size (*f* = 0.85) with a statistical power (1 − *β*) of 0.80 and a significance level (*α*) of 0.05 for one‐way ANOVA.

### 2.8 Surgical procedure and tissue collection

A rabbit model of scleral defects was developed following a previously reported method, with slight modifications to the surgical procedure.^43^ All surgical procedures were performed under general anesthesia. Zoletil (0.3 mL/kg; Virbac, Carros, France) and Rompun (0.2 mL/kg; Bayer Animal Health, Germany) were administered intramuscularly. Prior to surgery, the anterior segment of each eye was carefully examined under a surgical microscope to exclude pre‐existing ocular surface abnormalities or disease. The periocular skin and conjunctival sac were disinfected with 5% povidone‐iodine and thoroughly irrigated with sterile saline. Topical Alcaine® (proparacaine hydrochloride 0.5%; Alcon, Fort Worth, TX, USA) was subsequently applied to the ocular surface before the surgical procedure. A conjunctival incision was made on the nasal side of the eye, followed by a blunt dissection to expose the underlying sclera. A scleral defect measuring 10 mm × 10 mm and approximately 80% in depth was created using a surgical blade to produce a standardized lesion. Electrospun PCL/GE scaffolds (random, radial, or aligned) or the commercial collagen matrix Ologen™ were trimmed to appropriate sizes and sutured over the defect by using 8‐0 Vicryl sutures. For the aligned scaffolds, the fibers were oriented perpendicular to the corneal limbus. The conjunctiva was repositioned and secured with additional 8‐0 Vicryl sutures for complete closure. Postoperative care consisted of the topical administration of ofloxacin (Tarivid) and dexamethasone (Forus) ointments twice daily for 2 weeks. Clinical photographs were captured 2, 4, 6, 8, 12, 16, 20, and 24 weeks postoperatively to monitor wound healing and scaffold integration. At designated time points (6 or 24 weeks), the animals were euthanized under deep sedation, as confirmed by the loss of withdrawal reflexes. Potassium chloride (1 mL/kg) was administered intravenously to induce cardiac arrest. Both eyes were immediately enucleated and processed for further analysis.

The mRNA expression of IL‐1β, IL‐6, TNF‐α, and MMP‐9 in the tissue around the implant was measured by qPCR as described previously.^44^ RNA was isolated using a Tri‐RNA reagent (FAVORGEN, Ping‐Tung, Taiwan) and 1 μg of RNA was used to synthesize the complementary DNA (cDNA) using an RNA to cDNA EcoDry™ premix (TaKaRa, Shiga, Japan) according to the manufacturer's protocol. The relative mRNA levels were measured in ViiA7 (Applied Biosystems, Foster City, CA, USA) using a SYBR Green PCR Master Mix (Applied Biosystems). The sequences of the primers used in this study are shown in Table S2.

### 2.9 Imaging and histological processing

Following enucleation, the implantation sites were imaged using optical coherence tomography (OCT) with a Micron® IV system (Phoenix Research Labs, Pleasanton, CA, USA) to assess the in situ scaffold morphology and integration.^45^ Subsequently, the implanted regions were carefully dissected and fixed in 4% paraformaldehyde for 24 h at 4°C.^45^ The fixed tissues were dehydrated through a graded ethanol series, cleared with xylene, and embedded in paraffin. Paraffin blocks were sectioned at a thickness of 4 μm by using a rotary microtome. Histological evaluation was performed using hematoxylin and eosin (H&E) staining to assess general tissue morphology and Masson's trichrome staining to visualize collagen deposition and fibrous capsule formation.^46^

### 2.10 Evaluation of capsule formation and foreign body reaction

The fibrous capsule thickness was quantitatively analyzed on Masson's trichrome‐stained sections.^46^ Measurements were performed at more than 16 evenly distributed locations along the periphery of each implant cross‐section. Areas with processing artifacts were excluded, whereas vascular structures within the fibrotic capsule were included in the analysis. Foreign body reaction (FBR) was assessed using the eight‐tier histological grading system proposed by Harlim et al.,^47^ which classifies tissue responses based on inflammation, fibrotic changes, and the presence of foreign body giant cells (FBGCs). H&E and Masson's trichrome‐stained sections obtained at 6 and 24 weeks post‐implantation were examined for inflammatory cell infiltration, FBGC presence, and the extent of fibrosis. Each sample was categorized as Grade 1 (no reaction) to Grade 8 (extensive fibrosis with the absence of inflammatory cells and FBGCs).

To evaluate the ocular surface inflammatory response, grading of conjunctival hyperemia was performed at 6 and 24 weeks postoperatively using the standardized Efron scale (0 = normal, 1 = trace, 2 = mild, 3 = moderate, and 4 = severe).^48^ To quantitatively assess localized inflammation and foreign body responses, cell density in the peri‐implant tissue was quantified on H&E‐stained sections using QuPath, an open‐source digital pathology image analysis platform.^49^ Following established protocols for evaluating tissue response and cellularity around ocular biomaterials,^50^, ^51^ equal‐sized square regions of interest (ROIs; 200 × 200 μm each) were placed within the peri‐implant tissue or along the implant–tissue interface. For each specimen, measurements were taken at four points to determine the average value per eye. Automated cell detection was performed within each ROI, and the number of detected cells was normalized to the total analyzed tissue area and expressed as cells/mm^2^. This area‐normalized cell density served as a quantitative measure of peri‐implant tissue cellularity.

### 2.11 Statistical analysis

All quantitative data are presented as mean ± standard deviation (SD) from at least three independent experiments or biological replicates (*n* ≥ 3 per group). Group comparisons were performed using one‐way or two‐way ANOVA with Tukey's post hoc test, as appropriate. Paired Student's *t*‐test was used for within‐group comparisons before and after surgery. A *p*‐value <0.05 was considered statistically significant (*p < 0.05, **p < 0.01, ***p < 0.001, ****p < 0.0001).

## 3 RESULTS

### 3.1 Characterization of PCL/GE NFs

The morphology and orientation of the fabricated PCL/GE NFs were characterized using SEM imaging at different magnifications (2000× and 4000×), as shown in Figure 2a. The random scaffolds exhibited fibers intersecting at arbitrary angles with no predominant direction, creating a complex network structure. In contrast, the aligned scaffolds demonstrated a high degree of parallel fiber arrangement, with most fibers oriented along a single preferred direction. The radially aligned scaffolds exhibited an intermediate degree of orientation, with fibers exhibiting a discernible directional tendency, but with a greater angular distribution than the fully aligned scaffolds. The gelatin component within the nanofibers was chemically cross‐linked using glutaraldehyde vapor, which contributed to the preservation of the fiber morphology and structural integrity during handling and incubation. Quantitative analysis of the fiber orientation distribution, presented as histograms in Figure 2b, confirmed these visual observations. The random scaffolds exhibited a relatively flat distribution across all angles, indicating no preferential orientation. The radially aligned scaffolds exhibited a moderate peak in the orientation distribution, whereas the aligned scaffolds displayed a sharp narrow peak, demonstrating the highest degree of orientation uniformity. The average fiber diameter across all scaffold types was approximately 1 μm (Random: 0.98 ± 0.09 μm, Radially aligned: 0.94 ± 0.10 μm, Aligned: 1.05 ± 0.13 μm).

**FIGURE 2:**
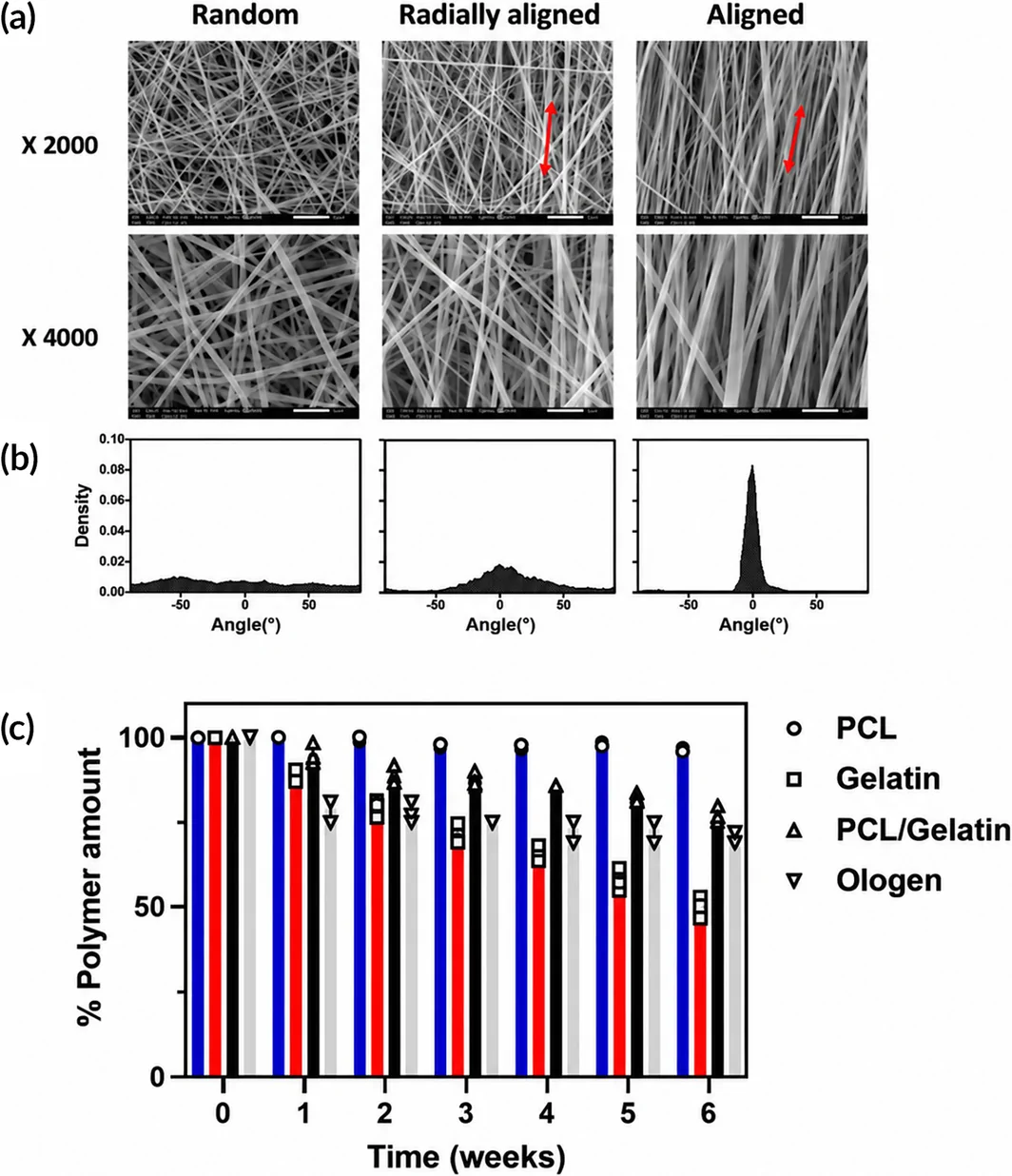
Physicochemical characterization of PCL/GE nanofibrous scaffolds. (a) SEM images of the three scaffold types: random, radially aligned, and aligned fibers (scale bars: 10 and 5 μm). (b) Quantitative analysis of fiber orientation using ImageJ confirmed increasing alignment from random to aligned scaffolds. (c) In vitro degradation profile of PCL/GE scaffolds and Ologen™ over 6 weeks. While all samples showed similar initial degradation, Ologen™ showed faster degradation after week 2 (*n* = 3).

FT‐IR analysis was performed to confirm the incorporation of gelatin into the PCL matrix (Figure S1). Pure PCL exhibited characteristic peaks at 2949, 2868, and 1720 cm^−1^, corresponding to asymmetric –CH_2_ stretching, symmetric –CH_2_ stretching, and carbonyl (C=O) stretching, respectively. Gelatin showed typical amide I and amide II bands at 1630 and 1520 cm^−1^, respectively.^52^, ^53^ In the PCL/gelatin scaffold, the characteristic peaks of both PCL and gelatin were clearly observed, confirming the successful incorporation of gelatin into the PCL matrix. XRD analysis was further conducted to evaluate the crystalline structure of the samples (Figure S2). Pure PCL exhibited a distinct diffraction peak at approximately 21.4°, indicating its semi‐crystalline nature.^54^ In the PCL/gelatin scaffold, this characteristic PCL peak was retained but showed reduced intensity and a broader profile, suggesting that the incorporation of gelatin influenced the crystalline structure of PCL.

To further evaluate the structural stability of the electrospun scaffolds, tensile mechanical testing was performed for the random, radially aligned, and aligned scaffold groups. Representative stress–strain curves are presented in Figure S3, and the corresponding mechanical parameters are summarized in Table S3. Fiber orientation significantly influenced the mechanical properties, with the aligned scaffold exhibiting the highest Young's modulus (26.58 ± 6.6 MPa) and ultimate tensile stress (5.51 ± 1.78 MPa), followed by the radially aligned and random scaffolds. Compared with previously reported values for native rabbit and human sclera, the electrospun scaffolds exhibited comparable or higher ultimate tensile strength, indicating sufficient mechanical robustness for scleral reconstruction.^42^

The porous architecture of the electrospun scaffolds was quantitatively analyzed by mercury intrusion porosimetry (Figure S4). All scaffold groups exhibited similarly high theoretical porosity (96.3%–96.9%) and comparable pore volumes, whereas the pore surface area decreased with increasing fiber alignment. Specifically, the random scaffold exhibited the highest pore surface area (421.0 ± 36.5 m^2^ g^−1^), followed by the radially aligned (344.5 ± 22.6 m^2^ g^−1^) and aligned scaffolds (273.8 ± 18.9 m^2^ g^−1^), while the pore volume remained comparable among the groups. These results indicate that fiber orientation primarily altered the internal pore architecture rather than the overall scaffold porosity.

The enzymatic degradation behavior of PCL, gelatin, PCL/gelatin, and Ologen (used as a control) was evaluated over 6 weeks in an enzyme‐containing solution to better reflect physiologically relevant degradation conditions, as shown in Figure 2c. PCL exhibited the highest stability, showing only minimal degradation throughout the test period. In contrast, gelatin underwent more rapid degradation, retaining approximately 50% of its initial mass after 6 weeks. The PCL/gelatin composite demonstrated intermediate behavior, maintaining about 77% of its initial mass, while the control group (Ologen) retained approximately 70%. SEM images of the scaffolds after 1, 2, 3, and 4 weeks of enzymatic degradation are shown in Figure S5. All scaffold groups retained their characteristic fibrous morphology throughout the degradation period. Moreover, the radially aligned and aligned scaffolds preserved their respective fiber orientation, indicating that the anisotropic architecture remained stable during enzymatic degradation. These results suggest that the incorporation of PCL enhances structural stability while still allowing partial biodegradation under enzymatic conditions.

### 3.2 Effect of PCL/GE nanofiber orientation on cell proliferation, alignment, and collagen production

Cell proliferation based on different orientations of the PCL/GE nanofibers was evaluated using the MTT assay (Figure 3a). On day 1, no significant differences were observed in cell proliferation among the three scaffolds (random, radially aligned, and aligned). However, from day 5, significant differences emerged in cell proliferation in the following order: aligned > radially aligned > random, with statistical significance. The absorbance values of the aligned and radially aligned groups plateaued between days 5 and 7 despite initially exhibiting significantly higher proliferation rates than those of the random group. This saturation is likely attributable to the early confluence of fibroblasts on the aligned scaffolds, where the directional fiber orientation facilitates efficient space utilization and promotes dense cell packing.

**FIGURE 3:**
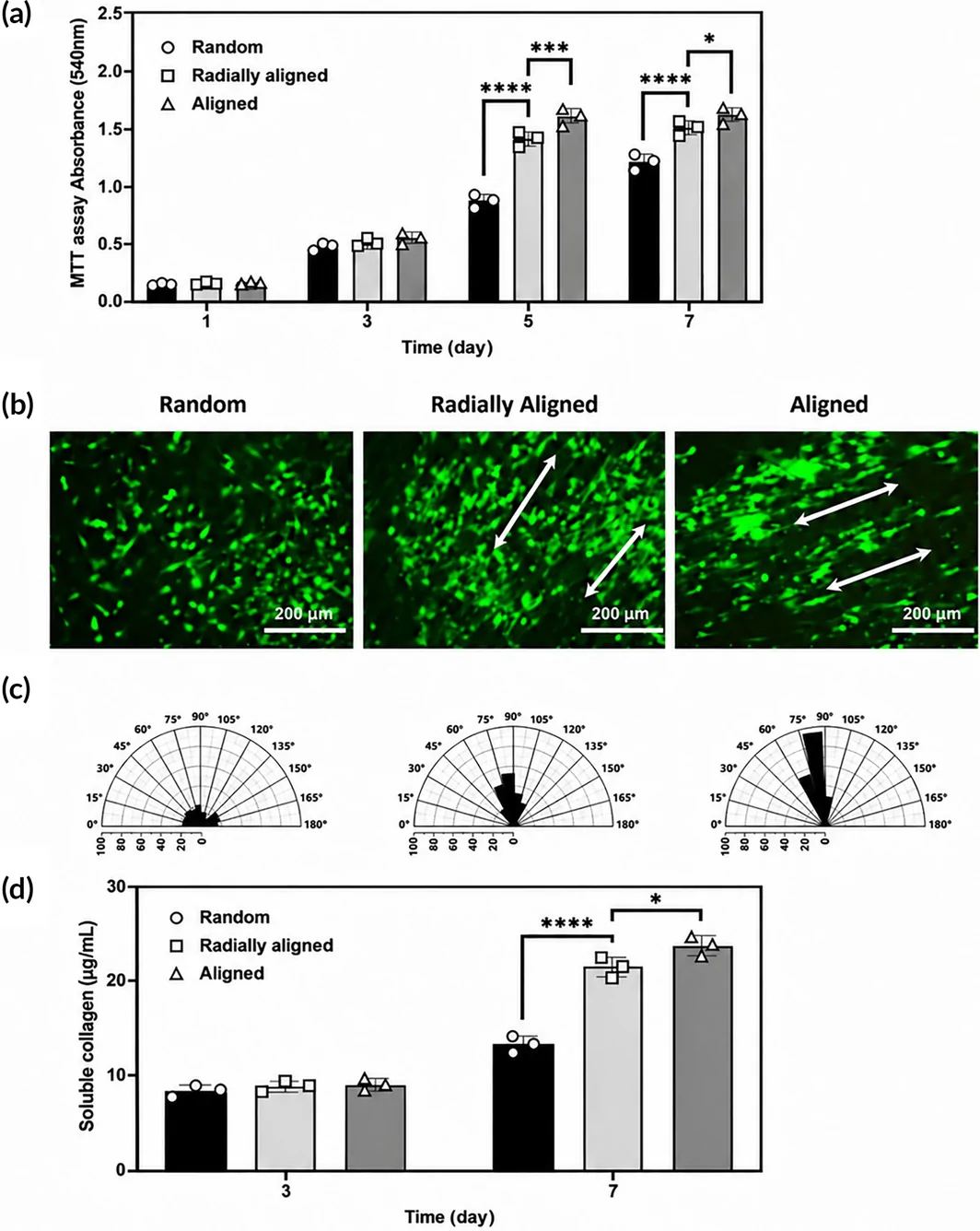
In vitro cellular responses of NIH3T3 fibroblasts cultured on PCL/GE scaffolds with different fiber orientations. (a) Cell proliferation assessed by MTT assay on days 1, 3, 5, and 7. Aligned scaffolds supported faster proliferation compared with random and radially aligned groups (*n* = 3). (b) Fluorescence live/dead staining on day 3 confirmed high cell viability (green: live, red: dead) across all groups, with cells visibly aligned along the nanofiber direction in the radially aligned and aligned scaffolds. (c) Quantitative analysis of cell orientation confirmed increased directional alignment in aligned scaffolds. (d) Collagen type I secretion measured on days 3 and 7 using Sircol assay showed higher production in aligned scaffolds by day 7 (*n* = 3). The statistical significance was determined using a two‐way ANOVA followed by Tukey's post hoc test (*p < 0.05, ***p < 0.001, ****p < 0.0001).

The cytotoxicity of eluates derived from PCL, gelatin, and PCL/gelatin scaffolds was evaluated using a cell viability assay (Figure S6). As shown, all groups exhibited cell viabilities comparable to the control, with values maintained near 100%. These results suggest that PCL, gelatin, and the PCL/gelatin composite do not induce appreciable cytotoxic effects under the tested conditions. A live/dead assay was performed to visualize the cell orientation and assess the cytotoxicity of the PCL/GE nanofibers (Figure 3b). Cells on the random scaffolds exhibited no specific directional orientation, whereas cells on both radially aligned and aligned scaffolds demonstrated distinct directional alignments. The predominant green fluorescence across all scaffold types, with minimal red fluorescence, further confirmed the excellent biocompatibility and negligible cytotoxicity of the PCL/GE nanofibers. Quantitative analysis of the cell orientation distribution using ImageJ (Figure 3c) confirmed that the aligned scaffold exhibited the highest degree of cell orientation.

Collagen type I secretion by NIH/3 T3 cells in the different PCL/GE nanofiber orientations was quantified using a collagen assay kit (Figure 3d). While no significant differences in collagen type I secretion were detected among the scaffolds on day 3, by day 7, secretion levels increased in the following pattern: aligned > radially aligned > random. This trend corresponded to the cell proliferation results, demonstrating that the aligned nanofiber structure more effectively promoted both cell proliferation and collagen secretion.

### 3.3 Early in vivo response (up to 6 weeks): Clinical observation, histology, and safety evaluation

To evaluate the short‐term tissue response following implantation, we monitored rabbit eyes for up to 6 weeks. Serial clinical photographs were obtained 2, 4, and 6 weeks postoperatively (Figure 4a). Both the aligned and radially aligned scaffolds exhibited smoother ocular surface healing, with reduced conjunctival congestion and surface irregularity compared with the random group (Figure S7a). Quantitative grading of conjunctival hyperemia using the Efron scale further confirmed that the aligned and radially aligned groups maintained significantly lower congestion scores than the random group at 6 weeks. Ologen™ showed the most favorable gross appearance, characterized by minimal vascularization and surface inflammation, likely owing to its rapid degradation and soft consistency, which reduced mechanical irritation.

**FIGURE 4:**
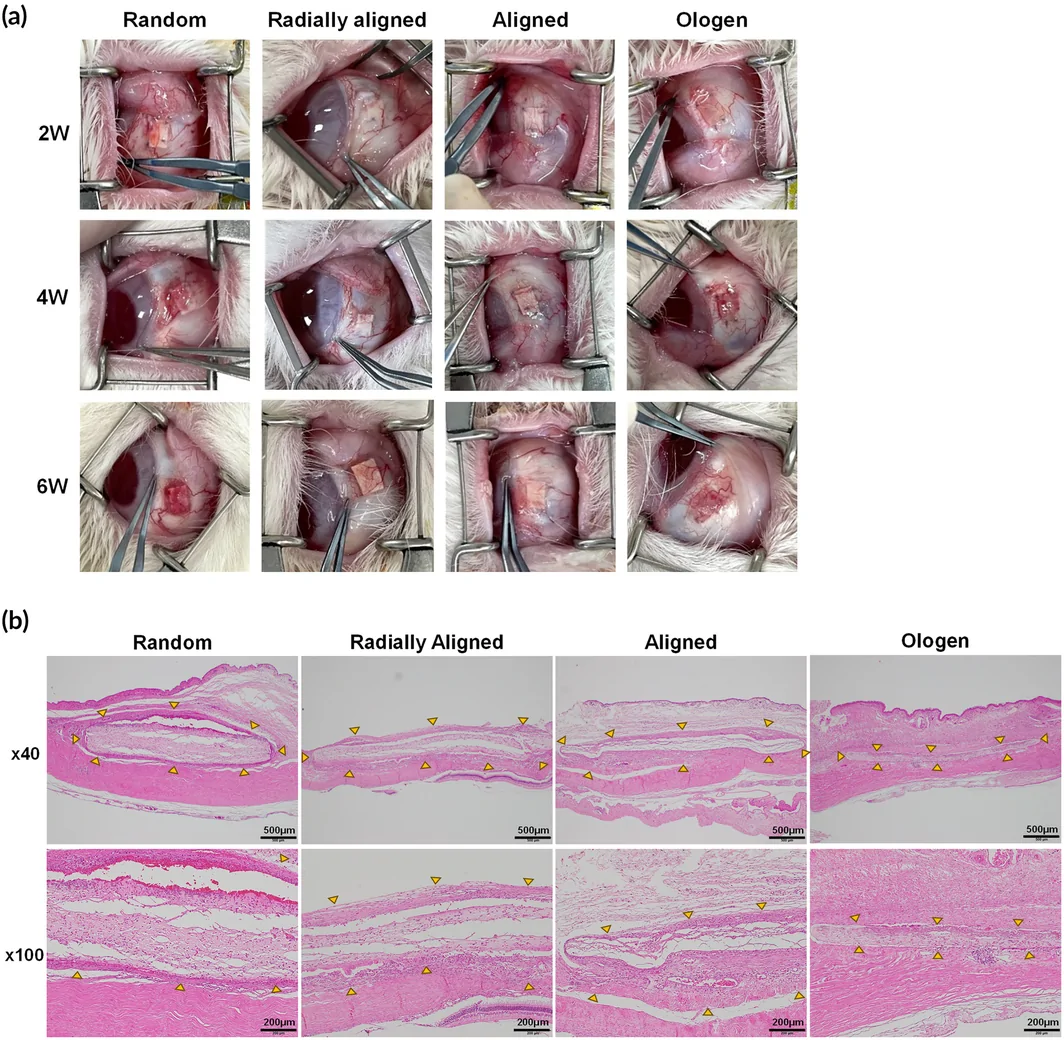
Short‐term in vivo evaluation of scaffold integration and inflammation (up to 6 weeks). (a) Anterior segment photographs of rabbit eyes at 2, 4, and 6 weeks post‐implantation show reduced vascularization and smoother healing in aligned and radially aligned groups compared with the random group. (b) H&E‐stained tissue sections at 6 weeks reveal inflammation and fibrosis in the random group, whereas aligned scaffolds and Ologen™ showed reduced host reaction. The arrowheads indicate scaffold location.

At 6 weeks, enucleated eyes were processed for histological evaluation by using H&E staining (Figure 4b). Scaffold remnants were visible in all groups (denoted by arrowheads). The random group exhibited prominent fibrosis and vascular proliferation around the implant, indicating a strong inflammatory response. In contrast, aligned and radially aligned scaffolds demonstrated a more organized host‐tissue interface with reduced inflammation and early matrix remodeling. Consistent with these findings, peri‐implant tissue cellular density analysis at 6 weeks revealed significantly lower inflammatory cell infiltration in the aligned and radially aligned groups compared to the random group (Figure S7b). Ologen™ exhibited minimal fibrotic changes and sparse vasculature, reflecting the mildest tissue response at this early stage.

To confirm the clinical safety of the PCL/GE scaffolds, intraocular pressure (IOP) and localized inflammatory responses were evaluated. All experimental groups maintained stable IOP within the physiological range up to 6 weeks post‐surgery, with no significant differences observed compared with the surgical control (Figure S8). Furthermore, qPCR analysis of pro‐inflammatory cytokines (IL‐1β, IL‐6, TNF‐α, and MMP‐9) in peri‐implant tissues showed no significant induction of inflammatory markers in any of the scaffold groups (Figure S9), indicating excellent in vivo biocompatibility.

### 3.4 Long‐term in vivo outcomes (up to 24 weeks): Structural integrity and histology

To assess the long‐term outcomes, clinical photographs were taken at multiple time points up to 24 weeks post‐implantation (Figure 5a). While Ologen™ implants were initially well tolerated, they were completely degraded by 24 weeks, leading to exposure of the underlying choroidal tissue and a darkened appearance at the defect site—indicative of both structural loss and poor cosmetic outcome. In contrast, PCL/GE scaffolds remained structurally intact throughout the observation period. Scaffolds with aligned or radially aligned fibers preserved a more regular ocular surface contour and exhibited minimal visible defect margins, suggesting favorable long‐term coverage and improved cosmetic results. Consistent with these gross observations, quantitative scoring using the Efron scale at 24 weeks revealed that both aligned and radially aligned groups maintained low conjunctival hyperemia scores, which were comparable to Ologen™ and significantly lower than those of the random group (Figure S7c).

**FIGURE 5:**
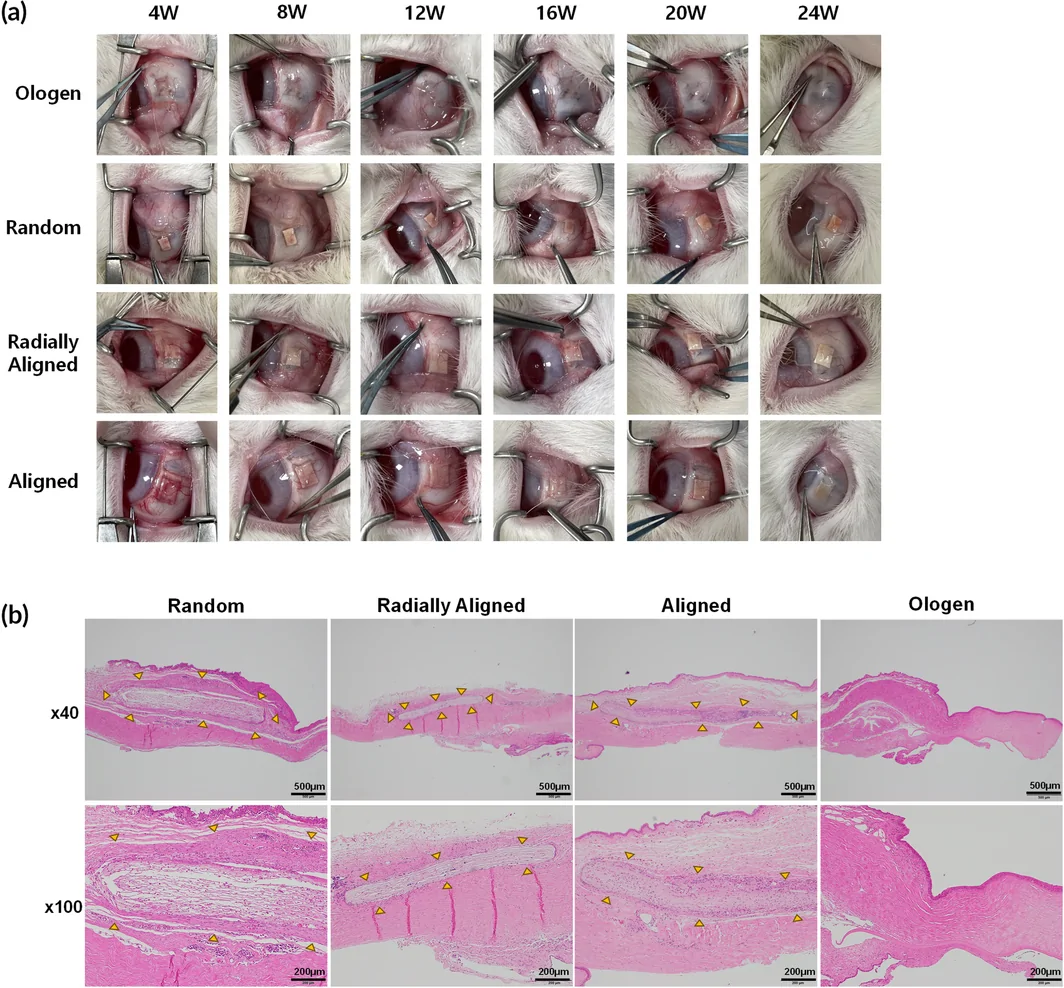
Long‐term in vivo evaluation of scaffold retention and structural integration (up to 24 weeks). (a) Serial clinical images from 4 to 24 weeks post‐implantation. Aligned scaffolds preserved surface contour and concealed the defects, whereas Ologen™ was fully resorbed by 24 weeks, exposing the underlying tissue. (b) H&E‐stained sections at 24 weeks showing intact scaffold structures in the anisotropic and random scaffolds, with better integration and tissue organization in the anisotropic scaffold. Ologen™ was completely degraded and absent in histological sections.

The histological analysis at 24 weeks (Figure 5b) supported these observations. H&E staining confirmed that both the aligned and radially aligned PCL/GE scaffolds maintained their architecture and were well integrated into the surrounding tissue. In comparison, the random group exhibited irregular collagen deposition and thicker fibrotic encapsulation despite structural persistence. This persistent chronic inflammation in the random group was further evidenced by a significantly higher peri‐implant inflammatory cell density compared to the radially aligned and Ologen™ groups (Figure S7d). Ologen™, consistent with gross findings, was fully resorbed and absent from tissue sections, confirming its limited durability for long‐term scleral repair.

### 3.5 Fibrosis and foreign body reaction evaluation by histology

Masson's trichrome staining and quantitative analysis of the fibrotic capsule thickness revealed clear differences in host responses among the scaffold types (Figure 6a,b). At 6 weeks, Ologen™ exhibited the thinnest fibrotic layer, likely owing to its rapid biodegradation and minimal mechanical presence. Among the PCL/GE groups, the aligned scaffold induced the least fibrous tissue formation, followed by the radially aligned and random scaffold groups. At 24 weeks, the radially aligned group exhibited the thinnest and most uniform fibrotic capsule, which was significantly thinner than those of both the aligned and random groups. The aligned group outperformed the random group, demonstrating the beneficial effect of fiber orientation in reducing chronic fibrotic remodeling. In contrast, the random group consistently exhibited the thickest and most irregular fibrous layers, indicating poor host integration. Ologen™ could not be analyzed at this time point because of its complete degradation. FBR scores, assessed via an eight‐tier classification (Figure 6c), showed no statistically significant differences between the groups at either 6 or 24 weeks post‐implantation.

**FIGURE 6:**
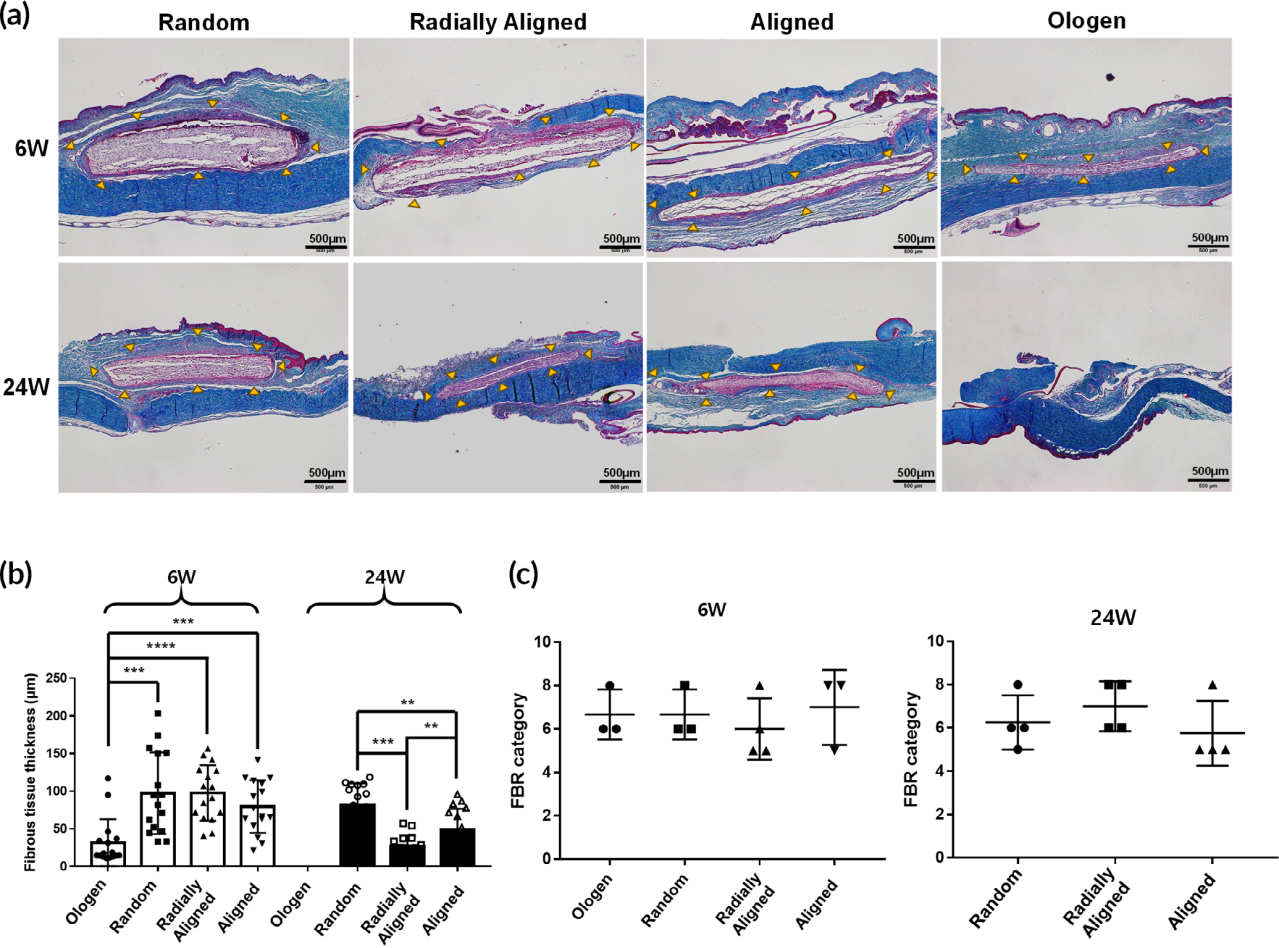
Histological analysis of fibrosis and foreign body response. (a) Masson's trichrome staining shows fibrous capsule formation at 6 and 24 weeks. (b) Quantification of capsule thickness indicates significantly thinner fibrotic layers in anisotropic scaffolds compared with random (*p < 0.05). (c) Foreign body reaction (FBR) scores assessed using an established 8‐point grading scale revealed no significant difference among groups. Statistical significance was determined using one‐way analysis of variance (ANOVA) followed by Tukey's honestly significant difference (HSD) post‐hoc test for multiple comparisons. Data are shown as mean ± SD (*n* = 4 per group).

### 3.6 OCT‐based assessment of structural integration

Optical coherence tomography (OCT) imaging was performed at 6 and 24 weeks post‐implantation to assess scaffold morphology and anatomical integration (Figure 7). At 6 weeks, the hyporeflective scaffold layers were visible in all groups (indicated by yellow arrowheads), although mild postoperative swelling partially obscured the posterior margins. The aligned and radially aligned PCL/GE scaffolds exhibited relatively uniform curvatures and well‐defined boundaries, suggesting favorable early integration. The random scaffold showed a more irregular thickness and curvature, indicating suboptimal structural conformity. The Ologen™ group also had a thin and homogeneous hyporeflective layer at this stage, reflecting partial degradation while retaining limited structural presence.

**FIGURE 7:**
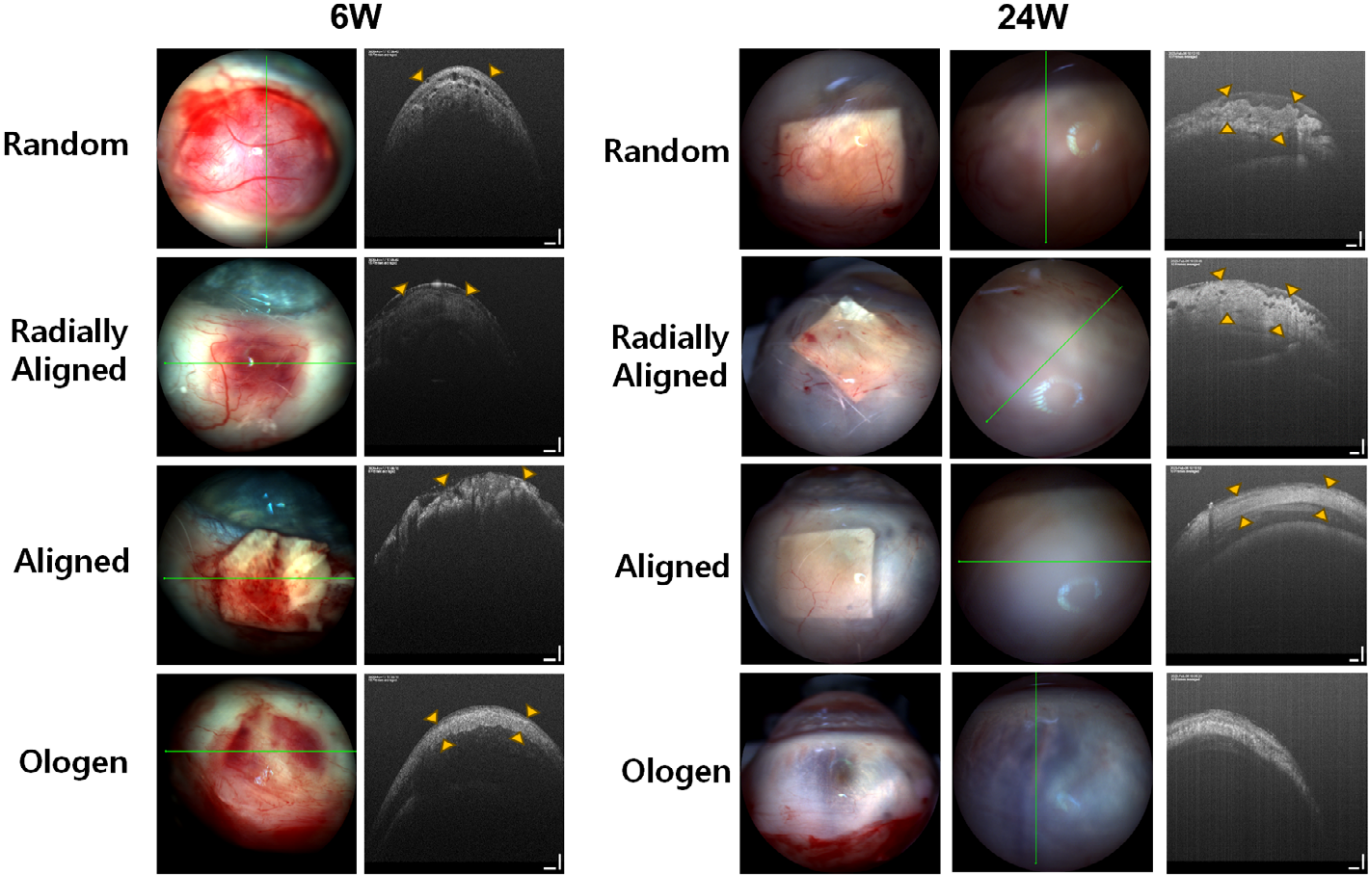
OCT‐based evaluation of scaffold integration. Cross‐sectional OCT images acquired at 6 and 24 weeks post‐implantation showed stable contour and retention of aligned scaffolds, whereas Ologen™ implants were no longer detectable by 24 weeks. The arrowheads indicate the location of the scaffold. Aligned scaffolds maintained a smooth and continuous profile, indicating favorable long‐term integration.

By 24 weeks, the distinct differences became more apparent. Both the aligned and radially aligned scaffolds preserved their shape and thickness with excellent conformity to the host sclera, indicating stable long‐term integration and minimal contraction. The random scaffold exhibited irregular contours, localized thinning, and signs of disorganization. In contrast, Ologen™ was no longer detectable by OCT, which was consistent with its complete degradation and lack of lasting structural support.

## 4 DISCUSSION

This study addressed the unmet need for durable and biocompatible scaffolds for scleral reconstruction. While collagen‐based materials such as Ologen™ are widely used, their rapid degradation limits long‐term structural support.^17^, ^46^, ^55^ To overcome this limitation, we developed electrospun nanofibrous scaffolds using a strategically designed blend of PCL and gelatin that leverages the complementary properties of synthetic and natural polymers.

Scleral defects arise from various pathological and iatrogenic conditions, including necrotizing or infectious scleritis and postoperative complications following ocular surgeries such as pterygium excision. Although relatively uncommon, these defects can lead to progressive thinning, uveal exposure, and even ocular perforation. A major limitation of current treatment strategies is the lack of durable “off‐the‐shelf” materials; for instance, collagen‐based matrices such as Ologen™ typically degrade within 3–6 months, which is insufficient for long‐term structural support. This mismatch between degradation and tissue remodeling often results in recurrent thinning and poor integration. From an engineering perspective, this reflects a fundamental limitation of existing materials, which primarily act as passive fillers without providing structural guidance for organized tissue regeneration.

The rationale for combining PCL with gelatin arises from the inherent limitations of each material when used alone. While PCL provides excellent mechanical strength and long‐term structural stability, its hydrophobic nature and lack of bioactive motifs limit effective cell–material interactions. In contrast, gelatin, a denatured form of collagen, offers superior biocompatibility and contains cell‐binding domains that promote cell adhesion and extracellular matrix production. Given that the native sclera is predominantly composed of type I collagen, the inclusion of gelatin creates a more biomimetic microenvironment that supports fibroblast attachment and tissue remodeling. However, gelatin alone degrades too rapidly to provide sustained structural support. Therefore, the hybrid PCL/gelatin system was designed to achieve a biphasic degradation profile, in which the gelatin component facilitates early cellular infiltration and matrix deposition, while the PCL matrix maintains long‐term mechanical integrity. This complementary behavior enables temporal coordination between biological activity and structural support, which is essential for effective scleral reconstruction.

Glutaraldehyde was selected as the crosslinker because it efficiently crosslinks free amino groups in gelatin, thereby improving the aqueous stability of gelatin‐containing electrospun fibers. It has also been widely used for crosslinking gelatin‐based electrospun nanofibrous scaffolds because of its high crosslinking efficiency, rapid reaction kinetics, and ability to stabilize the fibrous structure.^56^, ^57^ In the present study, vapor‐phase crosslinking was used to minimize fiber swelling, fusion, and structural distortion. Because crosslinking was performed on the cell‐free scaffolds prior to any biological contact, residual unreacted glutaraldehyde could be thoroughly removed by hydration and PBS washing before cell seeding or implantation. Consistent with this procedure, no appreciable cytotoxic or inflammatory effects were observed in both in vitro and in vivo evaluation.

The electrospinning technique enables the creation of ECM‐mimetic architectures with controllable fiber orientation, offering topographical cues for guided tissue regeneration. By evaluating three distinct fiber orientations (random, radially aligned, and aligned), we aimed to identify the scaffold designs that improve structural integration and minimize inflammation. The three scaffold groups were fabricated with comparable fiber diameters of approximately 1 μm so that fiber orientation, rather than fiber diameter, could be evaluated as the primary architectural variable. Electrospun fibers in this diameter range can provide effective topographical cues for fibroblast attachment and directional organization while maintaining a highly porous network suitable for cell infiltration and mass transport.^36^, ^58^ In the present study, all three scaffold groups exhibited similarly high porosity (>96%), further minimizing differences in overall porosity as a confounding factor. The three fiber architectures were selected to represent distinct patterns of structural guidance. Random fibers served as a conventional electrospun reference without preferential directional cues, whereas uniaxially aligned fibers provided strong contact guidance for directional cell organization and extracellular matrix deposition. Radially aligned fibers were included as a spatially varying anisotropic architecture capable of facilitating centripetal cell migration and potentially providing favorable conformity to the curved ocular surface. This design therefore allowed the specific influence of fiber organization on cellular and tissue responses to be evaluated while maintaining comparable fiber diameter and overall porosity among the groups. To our knowledge, this is the first study to directly compare anisotropic scaffolds with clinical standards in a scleral defect model, highlighting their translational potential.

As shown in Figure 2a, the desired fiber architectures (random, radially aligned, and aligned) were successfully obtained using controlled electrospinning techniques. Quantitative analysis (Figure 2b) confirmed clear differences in the fiber alignment among the groups. This ability to modulate scaffold anisotropy is significant because fiber orientation is known to influence cellular organization, matrix deposition, and ultimately, the quality of tissue regeneration. The degradation profiles indicate that incorporation of PCL significantly improved enzymatic stability (Figure 2c). Gelatin alone degraded rapidly, retaining only about 50% after 6 weeks. In contrast, the PCL/gelatin composite degraded more slowly, retaining approximately 77%, demonstrating that PCL effectively reduced the degradation rate. Moreover, the PCL/gelatin group showed higher degradation stability than Ologen (~70%), indicating enhanced structural integrity. This improvement is likely due to the hydrophobic and semi‐crystalline nature of PCL, which resists enzymatic degradation. Overall, the PCL/gelatin construct provides a more stable degradation profile than gelatin alone and Ologen, making it suitable for applications requiring prolonged support.

Considering that scleral fibroblasts are the primary producers of the ECM in the sclera, we used the NIH/3T3 fibroblast cell line to evaluate the in vitro performance of the PCL/GE nanofibrous scaffolds. As shown in Figure 3a, the MTT assay results demonstrated that cell proliferation was significantly enhanced on the aligned scaffolds compared with that on radially aligned and random scaffolds. This is likely owing to the physical guidance cues provided by the aligned fibers, which can direct cell spreading and migration along the fiber axis. Such topographical guidance promotes more efficient space utilization and facilitates denser cellular organization within the scaffold area.^59^, ^60^, ^61^, ^62^, ^63^ Live/dead staining confirmed these observations (Figure 3b,c). Cells cultured on aligned and radially aligned scaffolds exhibited elongation and orientation along the direction of the fibers, consistent with contact guidance behavior. In contrast, the cells on randomly oriented scaffolds showed no preferential alignment. Importantly, the minimal presence of dead cells across all groups indicated that the PCL/GE scaffolds had low cytotoxicity and good biocompatibility. Given that the sclera is primarily composed of type I collagen, the ability of fibroblasts to synthesize and secrete collagen is a key indicator of scaffold functionality in tissue regeneration. As shown in Figure 3d, collagen type I secretion by NIH/3T3 cells increased with increasing fiber alignment, with the aligned scaffold showing the highest levels. This trend is likely influenced by both higher cell proliferation and the elongated cell morphology induced by fiber orientation, which was shown to enhance ECM production in previous studies.^64^, ^65^, ^66^, ^67^ Although both aligned and radially aligned scaffolds provided directional cues, the aligned group showed slightly enhanced cellular responses overall. This may be attributed to the spatial consistency of the fiber orientation in the aligned scaffold, where unidirectional fibers offer uniform contact guidance across the entire surface. In contrast, the radially aligned scaffold presented a position‐dependent variation in the fiber direction, which may have led to moderate heterogeneity in cell alignment and ECM deposition. Nevertheless, the radially aligned scaffold also supported robust cell proliferation, alignment, and collagen production, suggesting its potential as a biologically favorable design for applications requiring curved or concentric tissue structures, such as the sclera.

In the present study, NIH/3T3 fibroblasts were used instead of primary ocular fibroblasts because they provide a well‐characterized and reproducible model for isolating the effects of scaffold topography on fibroblast proliferation, alignment, and collagen production. This approach was appropriate for our primary objective of comparing orientation‐dependent cellular responses under standardized conditions, while the overall study was focused on long‐term in vivo scaffold performance. Accordingly, the biological relevance of the scaffold designs was ultimately validated in a rabbit scleral defect model for up to 24 weeks, where the implants directly interacted with native ocular tissues. Future studies using primary rabbit or human scleral fibroblasts, or Tenon's fibroblasts, would further strengthen the tissue‐specific interpretation of these cellular responses prior to clinical translation.

The in vivo experiments demonstrated that PCL/GE nanofibrous scaffolds supported effective scleral tissue integration, with fiber orientation playing a key role in modulating host responses. At 6 weeks, as shown in Figure 4, both aligned and radially aligned scaffolds exhibited smoother ocular surfaces and less conjunctival congestion compared with the random group, suggesting improved early healing. Histological analysis confirmed that the aligned scaffolds induced milder inflammation and less vascular infiltration, whereas the random group exhibited significant fibrosis and neovascularization. Interestingly, Ologen™, a clinically used collagen‐based scaffold, exhibited the lowest inflammation and fibrosis at this early stage, likely owing to its soft texture and rapid biodegradability. However, its benefits were transient. By week 24 (Figure 5), the structural and histological differences among the groups became more pronounced. Ologen™ had fully degraded, resulting in exposure of underlying choroid and a visibly darkened defect site, compromising both cosmetic and mechanical outcomes. In contrast, all the PCL/GE scaffolds remained intact. The aligned group maintained the most stable surface contour, whereas the radially aligned scaffold preserved a well‐integrated dome‐shaped profile. However, random scaffolds showed an irregular morphology and less favorable integration.

Masson's trichrome staining further revealed that the fibrotic capsule thickness was lowest in the radially aligned group at 24 weeks, suggesting that this architecture may offer improved immunological compatibility and reduced long‐term fibrosis. Although the aligned scaffold supported a more uniform cell alignment and matrix organization, the radially aligned design provided comparable biocompatibility with the additional advantage of geometric conformity to the curved shape of the ocular surface (Figure 6a,b). FBR scores, evaluated using an established histological grading system, showed no statistically significant differences among the scaffold groups at either 6 or 24 weeks (Figure 6c). This indicated that all tested implants, including PCL/GE scaffolds with different fiber orientations and Ologen™, were generally well tolerated by host tissue without provoking excessive chronic inflammation or foreign body giant cell formation.

The reduced fibrotic response observed with the anisotropic scaffolds may be associated with topography‐mediated regulation of fibroblast organization. Aligned and radially aligned fibers provide contact‐guidance cues that promote directional cell organization and more organized matrix deposition, whereas random fibers provide heterogeneous directional cues. Previous studies have also reported that anisotropic topographies can modulate fibroblast activation and myofibroblast differentiation, which may partly explain the reduced fibrotic capsule formation observed in the aligned and radially aligned groups.^68^, ^69^, ^70^

OCT imaging highlights the importance of the scaffold architecture in maintaining long‐term structural stability. The ability of aligned and radially aligned scaffolds to preserve their shape over time suggests that fiber orientation helps resist contraction and conform to the natural curvature of the sclera, which is an essential factor in ocular applications where subtle surface distortions can affect function and appearance. These findings imply better integration and reduced mechanical mismatch in anisotropic scaffolds, potentially contributing to reduced chronic inflammation and fibrosis. In contrast, the complete disappearance of Ologen™ reflects a key limitation of fast‐degrading matrices: insufficient long‐term support despite early biocompatibility. Overall, the OCT results underscore that scaffold design influences not only cellular response but also anatomical retention, which is critical for successful scleral reconstruction.

These in vivo findings corresponded well with our in vitro observations, in which the aligned and radially aligned scaffolds promoted greater fibroblast proliferation, alignment, and collagen secretion. This consistency suggests that the favorable cellular behavior induced by fiber orientation in vitro translates into improved scaffold–host integration and immunological tolerance in vivo.

This study highlights several key aspects that differentiate our approach from existing scleral reconstruction strategies. While electrospun nanofibers have been widely explored in other ophthalmic applications, their use in scleral implants remains limited. Here, we demonstrate that electrospun PCL/gelatin scaffolds can be rationally designed to mimic the anisotropic architecture of native scleral tissue. By systematically comparing random, radially aligned, and aligned fiber orientations, we show that scaffold microarchitecture plays a decisive role in regulating fibroblast behavior, extracellular matrix deposition, and long‐term tissue integration. In particular, anisotropic fiber alignment contributed to reduced fibrotic encapsulation and improved structural conformity. Furthermore, unlike rapidly degrading collagen‐based materials such as Ologen™, the PCL/gelatin scaffold exhibits a biphasic degradation behavior, enabling initial cellular interaction through gelatin components while maintaining long‐term mechanical integrity via the PCL framework. Collectively, these findings highlight the importance of architectural design in biomaterial‐based scleral repair and underscore the translational potential of fiber‐aligned nanofibrous scaffolds for durable ocular reconstruction.

From a translational perspective, although the electrospinning process enables precise control over fiber architecture, further efforts are required to ensure reproducibility and scalability for clinical manufacturing. While consistent scaffold morphology and fiber alignment were achieved under controlled laboratory conditions, large‐scale production will require strict control of process parameters, environmental conditions, and batch‐to‐batch variability. In addition, standardization of scaffold thickness, fiber alignment, and mechanical properties will be essential for quality assurance and regulatory compliance. Another important consideration for clinical translation is the selection of appropriate sterilization and storage strategies. Although UV irradiation was used to sterilize the scaffolds in this preclinical study, we recognize that it is not suitable as a terminal sterilization method for clinical implants because of its limited penetration depth. Clinically established sterilization methods, such as gamma irradiation or ethylene oxide (EtO), should therefore be considered during future translational development. As sterilization conditions may influence the physicochemical and mechanical properties of polymer‐based scaffolds, an appropriate method should be selected with consideration of scaffold integrity and biological performance. In addition, appropriate packaging, storage conditions, and shelf‐life should be established to ensure long‐term product stability and reproducibility. Future studies will evaluate appropriate packaging and storage conditions and investigate whether prolonged storage affects the nanofibrous architecture, mechanical properties, and biological performance of the scaffolds. These evaluations will be important for ensuring product stability and reproducibility during manufacturing and clinical use.

## 5 CONCLUSION

This study provides the first evidence that controlled fiber orientation in electrospun PCL/gelatin nanofibrous scaffolds plays a decisive role in modulating scleral tissue regeneration, demonstrated through direct comparison with a clinically used collagen matrix and validated in a long‐term in vivo model. Scaffolds with directional fiber alignment enhanced fibroblast proliferation, ECM production, and tissue organization, while also reducing fibrotic encapsulation and preserving anatomical conformity to the ocular surface. Compared with randomly oriented scaffolds, anisotropic scaffolds showed more favorable tissue organization and reduced fibrotic encapsulation, while they provided markedly superior long‐term structural retention compared with Ologen™. They also maintained smoother surface contours and minimized choroidal show‐through, indicating improved cosmetic appearance. Importantly, these findings address critical limitations of current collagen‐based materials, including rapid biodegradation and inconsistent clinical availability, highlighting a clear unmet need for durable alternatives. These findings highlight the critical role of scaffold microarchitecture in modulating host response and guiding organized tissue regeneration. Overall, this work underscores the translational potential of aligned nanofibrous scaffolds as durable, biocompatible, and cosmetically favorable implants for ocular tissue engineering.

## AUTHOR CONTRIBUTIONS

**Ikhyun Jun:** Conceptualization; formal analysis; methodology; investigation; validation; visualization; funding acquisition; writing – original draft. **Seungmin Kang:** Formal analysis; investigation; validation; writing – original draft; methodology; data curation. **Taehyeon Kim:** Investigation; validation; visualization; writing – original draft. **Youngbin Seo:** Investigation; validation. **Hae‐sol Shin:** Validation; visualization. **Sejin Myeong:** Methodology; investigation. **David Myung:** Conceptualization; methodology. **Kyoung Yul Seo:** Conceptualization; methodology; resources; supervision; funding acquisition; writing – review and editing. **Won‐Gun Koh:** Conceptualization; funding acquisition; resources; supervision; writing – review and editing; methodology.

## CONFLICT OF INTEREST STATEMENT

The authors declare no conflicts of interest.

## Supporting information


**Figure S1.** Fourier‐transform infrared (FT‐IR) spectra of PCL, gelatin, and PCL/GE nanofibrous scaffolds.
**Figure S2:** X‐ray diffraction (XRD) patterns of PCL, gelatin, and PCL/GE nanofibrous scaffolds.
**Figure S3:** Tensile mechanical properties of electrospun PCL/GE nanofibrous scaffolds. Representative stress–strain curves of random, radially aligned, and aligned PCL/GE nanofibrous scaffolds obtained by uniaxial tensile testing.
**Figure S4:** Pore‐size distribution curves of random, radially aligned, and aligned electrospun PCL/GE nanofibrous scaffolds obtained by mercury intrusion porosimetry (top). Quantitative analysis of the theoretical porosity, pore volume, and pore surface area for each scaffold orientation is summarized in the table (bottom). Data are presented as mean ± standard deviation (*n* = 3).
**Figure S5:** SEM images of PCL/GE scaffolds after enzymatic degradation.
**Figure S6:** Cytotoxicity evaluation of scaffold extracts from PCL, gelatin, and PCL/GE nanofibrous scaffolds.
**Figure S7:** Conjunctival hyperemia score and inflammatory cell density analysis. (a, b) Clinical grading of conjunctival hyperemia using the Efron scale (a) and peri‐implant tissue cellular density analysis (b) at 6 weeks. For each specimen, the inflammatory cell density was measured at four points to determine the average value per eye. (c, d) Clinical grading of conjunctival hyperemia using the Efron scale (c) and peri‐implant tissue cellular density analysis (d) at 24 weeks. Statistical significance was determined using one‐way analysis of variance (ANOVA) followed by Tukey's honestly significant difference (HSD) post‐hoc test for multiple comparisons. Data are shown as mean ± SD (*n* = 4 per group; ns: not significant).
**Figure S8:** Comparison of intraocular pressure (IOP) before and after surgery across experimental groups. The intraocular pressure (IOP) was measured before (Pre) and 6 weeks after (Post) surgery in the Control, Random, Radially Aligned, and Aligned groups. No statistically significant changes in IOP were observed within any group following the procedure (paired *t*‐test, ns). Furthermore, there were no significant differences in IOP levels between the experimental groups (one‐way ANOVA, ns). Individual data points represent each eye, and the lines connect the pre‐ and postoperative measurements of the same eye. (ns: not significant).
**Figure S9:** Quantitative analysis of inflammatory cytokine expression at 6 weeks post‐implantation. To assess the biocompatibility of the scleral implants, the mRNA expression of pro‐inflammatory cytokines (IL‐1β, IL‐6, TNF‐α, and MMP‐9) was measured in the peri‐implant tissues using qPCR. At 6 weeks post‐surgery, all experimental groups (Random, Radially Aligned, and Aligned) showed no significant induction of inflammatory markers compared to the control group (one‐way ANOVA, ns). This suggests that the implants used for repairing the scleral defects did not elicit a chronic inflammatory response. All values were normalized to GAPDH and expressed relative to the control. (ns: not significant).
**Table S1:** Electrospinning parameters used for the fabrication of random, radially aligned, and aligned PCL/GE nanofibrous scaffolds.
**Table S2:** Primer sequences used in this study.
**Table S3:** Mechanical properties of electrospun PCL/GE nanofibrous scaffolds and comparison with native scleral tissue.

## Data Availability

The data that support the findings of this study are available from the corresponding author upon reasonable request.
