# Supplementary material for: Directionally aligned nanofibrous polycaprolactone/gelatin scaffolds as durable scleral implants for long‐term ocular reconstruction
Source: Bioeng Transl Med. 2026 Nov 10:e70196. Online ahead of print. doi: 10.1002/btm2.70196 (PMC13750463; doi:10.1002/btm2.70196)
Supplement: Supplementary file 1 [file BTM2-9999-e70196-s001.docx]

**[Supplementary Materials]**

**Directionally Aligned Nanofibrous Polycaprolactone/Gelatin Scaffolds as Durable Scleral Implants for Long-Term Ocular Reconstruction**

Ikhyun Jun ^a,b,1^, Seungmin Kang ^c,1^, Taehyeon Kim ^c, 1^, Youngbin Seo ^a^, Hae-sol Shin ^a^,

Sejin Myeong ^a,b^, David Myung ^d,e^, Kyoung Yul Seo ^a,b,^* and Won-Gun Koh ^c,^ *

^a^ The Institute of Vision Research, Department of Ophthalmology, Yonsei University College of Medicine, 50-1 Yonsei-ro, Seodaemun-gu, Seoul, 03722, South Korea

^b^The Corneal Dystrophy Research Institute, Department of Ophthalmology, Yonsei University College of Medicine, 50-1 Yonsei-ro, Seodaemun-gu, Seoul, 03722, South Korea

^c^Department of Chemical and Biomolecular Engineering, Yonsei University, 50 Yonsei-ro, Seodaemun-gu, Seoul 03722, South Korea

^d^Spencer Center for Vision Research, Byers Eye Institute, Department of Ophthalmology, Stanford University, Palo Alto, California 94304, USA

^e^Department of Chemical Engineering, Stanford University, Stanford, California 94305, USA

^1^ These authors contributed equally to this work.

*Corresponding author:

Won-Gun Koh, Ph.D.

Department of Chemical and Biomolecular Engineering, Yonsei University,

50 Yonsei-ro, Seodaemun-gu, Seoul 03722, Republic of Korea

Phone: 82-2-2123-5755, Fax: 82-2-312-6401

E-mail: wongun@yonsei.ac.kr,

Kyoung Yul Seo, M.D., Ph.D.

Department of Ophthalmology, Yonsei University College of Medicine

50-1 Yonsei-ro, Seodaemun-gu, Seoul 03722, Republic of Korea

Phone: 82-2-2228-3570, Fax: 82-2-312-0541

E-mail: [seoky@yuhs.ac](mailto:seoky@yuhs.ac)

**Supplementary materials contain 9 figures and 3 tables.**

**Figure S1**. Fourier-transform infrared (FT-IR) spectra of PCL, gelatin, and PCL/GE nanofibrous scaffolds.

**Figure S2.** X-ray diffraction (XRD) patterns of PCL, gelatin, and PCL/GE nanofibrous scaffolds.

**Figure S3.** Tensile mechanical properties of electrospun PCL/GE nanofibrous scaffolds.
Representative stress–strain curves of random, radially aligned, and aligned PCL/GE nanofibrous scaffolds obtained by uniaxial tensile testing.

**Figure S4.** Pore-size distribution curves of random, radially aligned, and aligned electrospun PCL/GE nanofibrous scaffolds obtained by mercury intrusion porosimetry (top). Quantitative analysis of the theoretical porosity, pore volume, and pore surface area for each scaffold orientation is summarized in the table (bottom). Data are presented as mean ± standard deviation (n = 3).

**Figure S5.** SEM images of PCL/GE scaffolds after enzymatic degradation.

**Figure S6.** Cytotoxicity evaluation of scaffold extracts from PCL, gelatin, and PCL/GE nanofibrous scaffolds.

**Figure S7.** Conjunctival hyperemia score and inflammatory cell density analysis. (a–b) Clinical grading of conjunctival hyperemia using the Efron scale (a) and peri-implant tissue cellular density analysis (b) at 6 weeks. For each specimen, the inflammatory cell density was measured at four points to determine the average value per eye. (c–d) Clinical grading of conjunctival hyperemia using the Efron scale (c) and peri-implant tissue cellular density analysis (d) at 24 weeks. Statistical significance was determined using one-way analysis of variance (ANOVA) followed by Tukey’s honestly significant difference (HSD) post-hoc test for multiple comparisons. Data are shown as mean ± SD (*n* = 4 per group; ns: not significant)

**Figure S8.** Comparison of intraocular pressure (IOP) before and after surgery across experimental groups. The intraocular pressure (IOP) was measured before (Pre) and 6 weeks after (Post) surgery in the Control, Random, Radially Aligned, and Aligned groups. No statistically significant changes in IOP were observed within any group following the procedure (paired t-test, ns). Furthermore, there were no significant differences in IOP levels between the experimental groups (one-way ANOVA, ns). Individual data points represent each eye, and the lines connect the pre- and postoperative measurements of the same eye. (ns: not significant)

**Figure S9.** Quantitative analysis of inflammatory cytokine expression at 6 weeks post-implantation. To assess the biocompatibility of the scleral implants, the mRNA expression of pro-inflammatory cytokines (IL-1β, IL-6, TNF-α, and MMP-9) was measured in the peri-implant tissues using qPCR. At 6 weeks post-surgery, all experimental groups (Random, Radially Aligned, and Aligned) showed no significant induction of inflammatory markers compared to the control group (one-way ANOVA, ns). This suggests that the implants used for repairing the scleral defects did not elicit a chronic inflammatory response. All values were normalized to GAPDH and expressed relative to the control. (ns: not significant)

**Table S1.** Electrospinning parameters used for the fabrication of random, radially aligned, and aligned PCL/GE nanofibrous scaffolds

**Table S2.** Primer sequences used in this study

**Table S3.** Mechanical properties of electrospun PCL/GE nanofibrous scaffolds and comparison with native scleral tissue

**Figure S1**. Fourier-transform infrared (FT-IR) spectra of PCL, gelatin, and PCL/GE nanofibrous scaffolds.

**Figure S2.** X-ray diffraction (XRD) patterns of PCL, gelatin, and PCL/GE nanofibrous scaffolds.

**Figure S3.** Tensile mechanical properties of electrospun PCL/GE nanofibrous scaffolds.
Representative stress–strain curves of random, radially aligned, and aligned PCL/GE nanofibrous scaffolds obtained by uniaxial tensile testing.

**Figure S4.** Pore-size distribution curves of random, radially aligned, and aligned electrospun PCL/GE nanofibrous scaffolds obtained by mercury intrusion porosimetry (top). Quantitative analysis of the theoretical porosity, pore volume, and pore surface area for each scaffold orientation is summarized in the table (bottom). Data are presented as mean ± standard deviation (n = 3).

**Figure S5.** SEM images of PCL/GE scaffolds after enzymatic degradation.

**Figure S6.** Cytotoxicity evaluation of scaffold extracts from PCL, gelatin, and PCL/GE nanofibrous scaffolds.

**Figure S7.** Conjunctival hyperemia score and inflammatory cell density analysis. (a–b) Clinical grading of conjunctival hyperemia using the Efron scale (a) and peri-implant tissue cellular density analysis (b) at 6 weeks. For each specimen, the inflammatory cell density was measured at four points to determine the average value per eye. (c–d) Clinical grading of conjunctival hyperemia using the Efron scale (c) and peri-implant tissue cellular density analysis (d) at 24 weeks. Statistical significance was determined using one-way analysis of variance (ANOVA) followed by Tukey’s honestly significant difference (HSD) post-hoc test for multiple comparisons. Data are shown as mean ± SD (ns: not significant)

**Figure S8.** Comparison of intraocular pressure (IOP) before and after surgery across experimental groups. The intraocular pressure (IOP) was measured before (Pre) and 6 weeks after (Post) surgery in the Control, Random, Radially Aligned, and Aligned groups. No statistically significant changes in IOP were observed within any group following the procedure (paired t-test, ns). Furthermore, there were no significant differences in IOP levels between the experimental groups (one-way ANOVA, ns). Individual data points represent each eye, and the lines connect the pre- and postoperative measurements of the same eye. (ns: not significant)

**Figure S9.** Quantitative analysis of inflammatory cytokine expression at 6 weeks post-implantation. To assess the biocompatibility of the scleral implants, the mRNA expression of pro-inflammatory cytokines (IL-1β, IL-6, TNF-α, and MMP-9) was measured in the peri-implant tissues using qPCR. At 6 weeks post-surgery, all experimental groups (Random, Radially Aligned, and Aligned) showed no significant induction of inflammatory markers compared to the control group (one-way ANOVA, ns). This suggests that the implants used for repairing the scleral defects did not elicit a chronic inflammatory response. All values were normalized to GAPDH and expressed relative to the control. (ns: not significant)

**Table S1.** Electrospinning parameters used for the fabrication of random, radially aligned, and aligned PCL/GE nanofibrous scaffolds.

| **Parameter** | **Random** | **Radially aligned** | **Aligned** |
| --- | --- | --- | --- |
| PCL/gelatin ratio | 1:1 | 1:1 | 1:1 |
| Polymer concentration | 5% / 5% (w/v) | 5% / 5% (w/v) | 5% / 5% (w/v) |
| Applied voltage | 12 kV | 12 kV | 12 kV |
| Flow rate | 1 mL/h | 1 mL/h | 1 mL/h |
| Tip-to-collector distance | 8 cm | 8 cm | 8 cm |
| Collector type | Stationary flat plate | Pin/ring collector | Rotating cylindrical collector |
| Collector speed | - | - | 900 rpm |

**Table S2.** Primer sequences used in this study

| Gene | Forward (5’→3’) | Reverse (5’→3’) | Product size (bp) |
| --- | --- | --- | --- |
| GAPDH | cgagatcccgccaacatcaa | agagttgtcatacttctcgtggt | 192 |
| IL-1β | gcaggtcttgtcagtcgttg | cgtactgcatcacactcaaga | 171 |
| IL-6 | agccccgacaaaaccctc | cgtcttcttctatcagccttgg | 175 |
| TNF-α | gaagagtccccaaacaacctc | cctccacttgcgggtttg | 124 |
| MMP-9 | cctgcgagtttccgttcatc | agacgaaggggaagacacac | 198 |

**Table S3.** Mechanical properties of electrospun PCL/GE nanofibrous scaffolds and comparison with native scleral tissue.

| **Type of sclera** | **Young’s modulus at 8% strain (MPa)** | **Ultimate stress (MPa)** | **Elongation at break (%)** |
| --- | --- | --- | --- |
| **Rabbit sclera*** | 4.46 ± 4.09 | 3.83 ± 2.87 | N/A |
| **Human sclera*** | 14.31 ± 8.56 | 4.45 ± 1.12 | N/A |
| **Random** | 19.57 ± 8.1 | 3.74 ± 1.81 | 62.07 ± 10.07 |
| **Radially aligned** | 22.45 ± 11.03 | 4.59 ± 1.99 | 59.83 ± 12.5 |
| **Aligned** | 26.58 ± 6.60 | 5.51 ± 1.78 | 53.60 ± 16.5 |

Young's modulus (determined at 8% strain) and ultimate tensile stress of random, radially aligned, and aligned PCL/GE nanofibrous scaffolds measured by uniaxial tensile testing. *Reported values for native rabbit and human sclera are included for comparison and were adapted from Zhang *et al.* (BioMed Research International, 2014, 194204).

**42.**	Zhang Y, Li Z, Liu L, Han X, Zhao X, Mu G. Comparison of Riboflavin/Ultraviolet-A Cross-Linking in Porcine, Rabbit, and Human Sclera. *Biomed Res Int.* 2014;2014:194204.
